# A Mobile App to Improve Self-Management of Individuals With Type 2 Diabetes: Qualitative Realist Evaluation

**DOI:** 10.2196/jmir.8712

**Published:** 2018-03-16

**Authors:** Laura Desveaux, James Shaw, Marianne Saragosa, Charlene Soobiah, Husayn Marani, Jennifer Hensel, Payal Agarwal, Nike Onabajo, R Sacha Bhatia, Lianne Jeffs

**Affiliations:** ^1^ Institute for Health System Solutions and Virtual Care Women's College Hospital Toronto, ON Canada; ^2^ Institute for Health Policy, Management, and Evaluation University of Toronto Toronto, ON Canada; ^3^ Keenan Research Centre Li Ka Shing Knowledge Institute St. Michael's Hospital Toronto, ON Canada; ^4^ American Academy of Nursing Washington, DC United States

**Keywords:** telemedicine, diabetes mellitus, self-management, qualitative research

## Abstract

**Background:**

The increasing use of Web-based solutions for health prevention and promotion presents opportunities to improve self-management and adherence to guideline-based therapy for individuals with type 2 diabetes (T2DM). Despite promising preliminary evidence, many users stop using Web-based solutions due to the burden of data entry, hidden costs, loss of interest, and a lack of comprehensive features. Evaluations tend to focus on effectiveness or impact and fail to evaluate the nuanced variables that may interact to contribute to outcome success (or failure).

**Objective:**

This study aimed to evaluate a Web-based solution for improving self-management in T2DM to identify key combinations of contextual variables and mechanisms of action that explain for whom the solution worked best and in what circumstances.

**Methods:**

A qualitative realist evaluation was conducted with one-on-one, semistructured telephonic interviews completed at baseline, and again toward the end of the intervention period (3 months). Topics included participants’ experiences of using the Web-based solution, barriers and facilitators of self-management, and barriers and facilitators to effective use. Transcripts were analyzed using thematic analysis strategies, after which the key themes were used to develop statements of the relationships between the key contextual factors, mechanisms of action, and impact on the primary outcome (glycated hemoglobin, HbA_1c_).

**Results:**

Twenty-six interviews (14 baseline, 12 follow-up) were completed with 16 participants with T2DM, and the following 3 key groups emerged: the easiest fit, the best fit, and those who failed to activate. Self-efficacy and willingness to engage with the solution facilitated improvement in HbA_1c_, whereas competing priorities and psychosocial issues created barriers to engagement. Individuals with high baseline self-efficacy who were motivated, took ownership for their actions, and prioritized diabetes management were early and eager adopters of the app and recorded improvements in HbA_1c_ over the intervention period. Individuals with moderate baseline self-efficacy and no competing priorities, who identified gaps in understanding of how their actions influence their health, were slow to adopt use but recorded the greatest improvements in HbA_1c_. The final group had low baseline self-efficacy and identified a range of psychosocial issues and competing priorities. These participants were uncertain of the benefits of using a Web-based solution to support self-management, ultimately resulting in minimal engagement and no improvement in HbA_1c_.

**Conclusions:**

Self-efficacy, competing priorities, previous behavior change, and beliefs about Web-based solutions interact to determine engagement and impact on the clinical outcomes. Considering the balance of these patient characteristics is likely to help health care providers identify individuals who are apt to benefit from a Web-based solution to support self-management of T2DM. Web-based solutions could be modified to incorporate the existing screening measures to identify individuals who are at risk of suboptimal adherence to inform the provision of additional support(s) as needed.

## Introduction

The number of people affected with diabetes worldwide has increased from 171 million to 422 million between 2000 and 2017, making it one of the most costly and devastating chronic diseases [1]. In Ontario, Canada, the prevalence of diabetes increased by 69% from 1995 to 2005 [2], exceeding the global increase of 60% previously projected to occur between 1995 and 2030 [3,4]. This dramatic rise is attributed to new cases of type 2 diabetes mellitus (T2DM) [5,6], driven by increasing rates of obesity [7,8]. Physical inactivity, smoking, alcohol consumption, and poor dietary habits have also been identified as risk factors that significantly increase an individual’s risk of developing T2DM [9]. Long-term complications include retinopathy, nephropathy, autonomic neuropathy leading to cardiovascular symptoms, and peripheral neuropathy with a risk of foot ulcers and amputations [5]. Most notably, individuals who have T2DM are twice as likely to die over a 12 year follow-up period compared with those without diabetes [10,11].

Given the severity and nature of disease progression, a cornerstone of clinical management is the process of teaching individuals how to manage their diabetes. An individual’s attitudes, beliefs, and knowledge about diabetes may affect diabetes self-management, including their adherence to prescribed pharmacotherapy [12,13], highlighting the need for individualized, patient-centered approaches. T2DM education and self-management education is a cost-effective approach [14] and has a direct impact on patients’ glycemic control [15]. Duration of contact between educator and patient has been noted to be a significant predictor of improved glycemic control in this population, underscoring the role of social support [15].

Despite advances in diabetes treatment and education, adherence to diabetes self-management regimens continues to be the most significant determinant of achieving clinical targets (ie, glycemic control) [16,17]. Barriers to diabetes management include individual attitudes and beliefs, knowledge, financial constraints, and social support [18-21]. Given the prevalence of mobile devices [22] and the increasing use of Web-based solutions for health prevention and promotion [23,24], mobile phone apps have emerged as a potential solution to improve self-management and adherence to guideline-based therapy due to their accessibility, low cost, and interactive potential [22]. These apps may include a range of features, including blood glucose monitoring, medication tracking, exercise tracking, and dietary management [25,26]. Although preliminary evidence looks promising [27-29], many app users stop using health apps due to high burden of data entry, hidden costs, loss of interest, and a lack of comprehensive features in a single solution [23,30-32]. Formal evaluations of Web-based solutions tend to focus on the effectiveness or impact and fail to evaluate the nuanced variables that may contribute to success (or failure) [33]. To address this gap in the literature, we conducted a qualitative realist evaluation as part of a larger randomized trial of a mobile-based self-management app to improve self-management in T2DM [34]. The objective was to identify key combinations of contextual variables and mechanisms of action that explain for whom the app worked best and in what circumstances.

## Methods

### Study Design

A qualitative realist evaluation [35] was embedded as part of a larger pragmatic, randomized, wait-list control trial to evaluate whether and how a mobile app designed to improve self-management and experience of care among patients with T2DM [34]. Realist evaluation is a methodology used to unpack the black box of implementation [36] by purposively examining the actions required by those involved in an intervention to ensure its success, including participants and those involved in implementation. This methodology enables a rigorous assessment of the contextual influences and strategies by which the intervention is adopted or rejected, enabling and understanding of how and why the implementation succeeds or fails. Specifically, a realist evaluation provides an explanation for why study outcomes occur, involves multimethods involving quantitative and qualitative approaches, and uses a theory-driven approach that guides the study design [35].

Trial participants were randomized to either an immediate treatment group (ITG) or a wait-list control group (WLC). The ITG group began using the mobile app immediately for a duration of 3 months. A series of quantitative outcomes were collected as part of the trial and are outlined in the original protocol [34]. Of particular relevance to this realist evaluation were the following 2 outcomes: glucose control (measured by HbA_1c_), and the Problem Areas in Diabetes 5 (PAID 5) [37], a measure of disease-specific self-efficacy that emphasizes well-being. Self-efficacy refers to an individual’s belief in his or her capability to achieve a given objective, which is a well-established mediator of health behaviors [38].

The intervention was implemented and supported by the Ontario Telemedicine Network (OTN), a nonprofit, government-funded organization and the largest provider of telemedicine services in the province of Ontario [39]. The protocol received ethics approval from Research Ethics Boards at participating institutions, including Women’s College Hospital, St. Joseph’s Care Group, North York General Hospital, and William Osler Health System. The larger trial is registered on ClinicalTrials.gov (NCT02813343).

### Intervention

The intervention is a commercially available app designed to serve as a Web-based coach for patients with T2DM (WellDoc Bluestar allows participants to enter a range of baseline clinical information, in addition to ongoing data related to diabetes management, including blood glucose values, daily medications, food intake, and activity levels). The app analyzes inputted data to provide tailored messaging to coach participants with respect to their diabetes management. Participants also had the option of emailing a SMART Visit report to a member of their care team via the app, which provides them with an overview of inputted data over a period specified by the participant. At the time of this study, the app did not include secure messaging with providers or social functionality to connect participants with one another. The mobile app has been shown to improve glycemic control (as represented by reduction in levels of HbA_1c_) in other contexts and settings [28,40].

The Web-based solution was implemented across 3 Diabetes Education Centers selected by the OTN. These sites were the Diabetes Health Centre in Thunder Bay, the Diabetes Education Center at North York General Hospital, and 2 Diabetes Education Centers belonging to the William Osler Health System. The OTN provided each site with funding for a site project coordinator who was responsible for recruiting participants and introducing them to the app. More than 4500 patients are seen across these sites annually, representing a socially and ethnically diverse group of individuals with diabetes. Each site serves distinct populations, including a large Indigenous population in Thunder Bay and visible minorities and newcomers in the William Osler Health System.

### Recruitment

Potential interview participants were recruited from the ITG group to ensure maximum potential for exposure to the app. The complete recruitment strategy has been described previously [34]. One-on-one, semistructured telephonic interviews were conducted, with questions guided by the principles of Realist Evaluation [35]. Topics include participants’ experiences of learning about and using the technology, barriers and facilitators of self-management, and barriers and facilitators to effective use (refer to Multimedia Appendix 1 for interview questions). Participants were interviewed at baseline and again toward the completion of the intervention period (3 months).

### Data Analysis

Interviews were conducted by an experienced qualitative researcher, audio-recorded, and transcribed by a third party. Transcripts were analyzed using thematic analysis strategies [35,41], which included identifying key themes that demonstrate important contextual influences and mechanisms of action for the Web-based solution in real-world health care settings. Recruitment continued until data saturation was reached. A minimum of 2 reviewers independently coded all transcripts using an open coding process. Following the first 5 interviews, a coding schema was created to guide the analysis of the subsequent interviews. Open coding was applied throughout the analysis for content that did not fit within the existing coding schema. Consolidation of codes was achieved through consultation with a third reviewer. There were no disagreements with respect to coding.

Several strategies were employed to ensure credibility of the data, such as using multiple sources of data, having key collaborators participate in the triangulation analysis and the return of findings (construct and external validity), examining points of convergence and divergence within and across cases (internal validity through cross comparative analyses), and having a stepped analysis process whereby there is an initial independent review of the data by 3 reviewers (LD, MS, and LJ) who then met to reach consensus on the common themes (reliability) [42].

After the thematic analyses of all qualitative data had been completed, the key themes identified were used to develop statements of the relationships between (1) key contextual factors, (2) the mechanisms by which they affect the implementation of the Web-based solution, and (3) the impact on the outcomes of the intervention itself (in Realist Evaluation these statements are referred to as *Context-Mechanism-Outcome [C-M-O] Configurations*) [35].

## Results

### Findings

A total of 26 interviews (14 baseline, 12 follow-up) were completed with 16 participants with T2DM across the 3 sites. Of the 14 participants who completed the baseline interview, 3 had dropped out and 1 was unavailable at follow-up; therefore, additional 2 participants were recruited to achieve data saturation at follow-up. Characteristics of patient participants are shown in Table 1. Patient participants were grouped according to their primary outcome from the trial data (HbA_1c_) and self-reported level of engagement with the app to describe C-M-O configurations. From the data, 3 groups emerged that are described in Table 1 (see Table 1 for a summary of participant characteristics by group).

### Group 1: The Easiest Fit—Engaged Early Adopters

The first C-M-O configuration concerns a group of individuals who had a high level of self-efficacy when self-managing their T2DM before the intervention, took ownership for their actions and were motivated to change, reported no competing priorities, were keen to engage with mobile technology to help support self-management, and were newly diagnosed with T2DM (3-9 months). A high level of preintervention self-efficacy was illustrated by having a positive mindset and reported behavior change and HbA_1c_ levels that were improving before enrolment in the study. These actions included increased physical activity, portion control, avoiding high-fat foods, and medication adherence.

**Table 1:** Patient participant characteristics.

Characteristics		Group 1: Engaged, early adopters (n=4)	Group 2: Engaged, slow adopters (n=4)	Group 3	
				Low engagers (n=5)	Dropouts (n=3)
Age in years, mean (range)		57 (51-63)	59 (49-67)	42 (32-52)	45 (37-49)
Sex (male:female)		4:0	2:2	2:3	2:1
Time since T2DM^a^ diagnosis, mean (range)		6 months (3-9 months)	19 years (9-26 years)	6 years (4 months-13 years)	3 years (3 months-27 years)
Complications from T2DM, n		1	3	2	4
**Site, n**					
	NYGH^b^	0	3	1	1
	Thunder Bay	2	1	1	2
	WOHS^c^	2	0	3	0
**Marital status, n**					
	Married	3	2	2	-
	Divorced	1	2	-	1
	Common law	-	-	1	1
	Single	1	1	2	-
**Ethnic background, n**					
	White	2	3	4	-
	First Nations	-	-	-	2
	African American	-	1	1	1
**Highest education, n**					
	High school	-	3	-	-
	University	1	-	2	2
	Postgraduate	3	1	3	1
**Type of therapy, n**					
	Oral	4	-	3	1
	Insulin	-	1	1	-
	Combination	-	3	1	2

^a^T2DM: type 2 diabetes.

^b^NYGH: North York General Hospital.

^c^WOHS: William Osler Health System.

Individuals in this group also exhibited proactive, information-seeking behavior to help them manage their T2DM:

I started looking up what information was on [the Diabetes Canada website], found out about what the daily types of meals you should have to control your blood sugar, and I immediately started following that.

SITE A05

Participants described various aspects of their identity that fuelled their motivation to change, which involved social activities, occupation, family, and overall quality of life. Narratives outlined feelings of accountability to oneself and taking ownership for individual actions:

Well it’s to my own benefit right, you know, if I do it then I’m the one that hopefully gets rewarded, it’s self-serving in a lot of places for me right...I, you know, I want to still have life left and do things I want to do.

SITE A05

Unlike participants in other groups, high engagers did not identify competing priorities that interfered with their ability to manage their T2DM. Instead, they described integrating management strategies into the existing schedules and routines. The majority described strong support networks at home that helped them adhere to their prescribed diet and activity recommendations. Only 1 individual described his social network and relationship with his family, but did not link these relationships to his self-management behavior. The study project coordinator, who was responsible for introducing the app to participants, was also viewed as a source of self-management support at 1 particular site:

We kinda have a kind of conversation when we get together...more like a friendly visit instead of an ‘oh I have to report to the nurse’...Yeah, I think I like her, and I know that I can just phone her up, she told me that, just phone her up, I mean if you need any advice or whatever–so that was nice.

SITE B06

These individuals were interested in using mobile technology to improve their health and enthusiastically engaged with the app immediately and consistently thereafter. Engaging with the intervention led to the activation of several mechanisms of change for these individuals. The data entry requirement of the app reinforced and strengthened the pre-existing accountability to self. Individuals described feeling “grounded” and “honest,” and explained how visualizations of their data helped to keep them on track and triggered a greater commitment to self-management:

[The application] keeps me grounded and keeps me honest. Even though you can put whatever you want in there and say ‘Oh yeah I’m having 10 slices of pizza and you only put in 1’...But it keeps me honest...It keeps me on track.

SITE B01

Data entry led to positive performance feedback that further enhanced the individual’s ability to self-monitor. Visual feedback displaying desired outcomes “reinforced positive behaviors” and encouraged participants to continue making informed choices and monitoring outcomes. These mechanisms interacted with each individual’s context to produce improvements in their primary clinical outcome (HbA_1c_) and their overall ability to manage their T2DM. These self-reported improvements were supported by the quantitative outcomes collected as part of the larger trial (refer to Table 2).

My A_1c_ is now pretty perfect...In April it was 10.4 and uhm, in November it was 6.5.

SITE B06

All individuals in this group highlighted that, in addition to the primary outcomes highlighted above, engaging with the app helped increase their awareness of their T2DM and the impact of stress and diet on their glucose readings. This increased awareness translated into increased self-efficacy with respect to self-managing T2DM.

Before I was clueless, not totally clueless, I just thought it was the sugary thing, I didn’t know how much the carbs got involved, and the fats.

SITE B 06

### Group 2: The Best Fit—Engaged Slow Adopters

The second group of C-M-O configurations concerns a group of individuals who had moderate self-efficacy in terms of self-managing their T2DM before the intervention, described an incomplete understanding of how actions influence their health and why, reported no competing priorities, were open to the idea of using mobile technology to support self-management, and had a long-standing diagnosis of T2DM.

Participants in this group described frustrations with the episodic nature of managing their condition and repeated unsuccessful attempts to “fine-tune” their self-management strategy. Despite a partial understanding of strategies to manage T2DM, these individuals strongly expressed their desire to fill these knowledge gaps. As a result, participants in this group identified the need for a specific solution that targeted their ability to achieve a more nuanced understanding of their T2DM (9-26 years):

You just go through stages of depression, you go through stages of anger, depression, denial, and then you sort of wake up and say, ‘Ok, I’ll just keep trying.’ And then you try again and you’re good for a couple years and then something happens, you get sick or, and there sometimes it’s discouraging because...it doesn’t matter what you do. I can take all the insulin I want but for me, when I’m sick, I can’t get my blood sugar down.

SITE A02

I have a feeling that my readings after dinner are still too high, but because I can’t break it down I don’t have the motivation to take the last step which is to write down everything you eat at dinner for the next three weeks and how much insulin you took so then we can address the little problems.

SITE C02

Participants described how the mobile app met these needs, which varied depending on the individual participant. Overall, the intervention enabled participants to track inputs such as diet and stress and their impact on a specific outcome, blood glucose levels. This mechanism of performance feedback increased participant awareness around which actions influenced their disease management:

With this, looking at whether I’m putting in my carbs and that, thinking ‘Ok, well I can only have...this. Yeah. That’s all I can have.’ So I think it’s made me more think about the fact that I can only eat so much and before it was just like, ah the heck with it.

SITE B02

Data tracking and trend visualization increased participants’ sense of accountability for their actions. Participants described their new-found accountability both to themselves and their health care providers, to whom they were very well connected. Data visualization enabled participants to see positive results in-between health care provider visits and encouraged incremental increases in engagement with the app over time:

The app helps me, you know, to be testing my blood and recording it and seeing any positive changes that I’m making. And the positive changes in turn help to sort of encourage me to continue it...so it’s like a circle if you will.

SITE C03

Real-time, nuanced performance feedback displayed glucose readings alongside symptom and dietary inputs and organized inputs by time of day. This enabled participants’ self-monitoring ability. In the case of this group, feedback displaying desired outcomes “drew attention to positive behaviors” and encouraged participants to continue making healthy decisions and monitoring outcomes. These mechanisms interacted with each individual’s context to produce improvements in their primary clinical outcome (HbA_1c_) and their overall ability to manage their T2DM.

**Table 2:** Mixed-methods results matrix.

Contextual variables, mechanism of action, and outcome			Group 1: High engagers, early adopters (easiest fit)	Group 2: High engagers, slow adopters (best fit)	Group 3	
					Low engagers (failed to activate)	Dropouts (failed to meet needs)
**Contextual variables**						
	Preintervention self-efficacy		High (numerous examples of positive behavior change with improved outcomes)	Moderate (some evidence of positive behavior change with variable impact in outcomes)	Low (no evidence of behavior change)	Low (no evidence of behavior change)
	Individual identity (includes affect)		New diagnosis	Longstanding diagnosis	Managing T2DM^a^ is a struggle and burden	Prospect of managing T2DM competing with psychological issues
			Positive attitude toward life and disease management	Episodic nature of T2DM management leads to frustrations	Described negative emotions (eg, anxiety, depression, anger)	Described negative emotions (eg, anxiety, depression, anger)
			Strong identity that serves as motivation to maintain “healthy” life			
	Health beliefs		Proactive, seeks out information	Partial understanding of strategies to manage T2DM	Report barriers to managing T2DM (eg, feelings of deprivation)	Not motivated to better manage T2DM
			Takes ownership	Uncertainty around the impact of certain individual actions	Lack of recognition around proper management	
			Accountable to self			
	Support system		Support at home facilitates adherence to diet and recommendations	Well-connected to health care providers for support	No support identified	No support identified
			Project coordinator identified as a source of support			
	Competing priorities		None described	None described	Multiple (family, school, work)	Multiple (family, school, work)
Mechanism of action			Performance feedback facilitates self-monitoring	Improved ability to track outcomes increased awareness	Preliminary signs that the app had potential	Participants did not engage with the mobile app
			Data entry reinforces accountability to self	Improved understanding of how individual actions affect T2DM	Mobile app failed to activate mechanisms of change in context	
			Positive outcomes reinforce behavior	Data visualization increased accountability for individual actions		
**Outcomes**						
	**HbA_1c_^b^, mean (range)**					
		Baseline	7.5% (6.2-9.9)	10.0% (8.7-11.1)	8.7% (6.9-10.6)	10.7% (9.7-12.6)
		3 months	6.0% (5.2-6.5)	8.3% (7.4-9.2)	8.8% (7.3-10.3)	N/A^c^
	**PAID5^d^, mean (range)**					
		Baseline	4.3 (1-8)	8.5 (3-14)	10.0 (3-16)	10.0 (5-15)
		3 months	3.5 (0-10)	7.3 (2-15)	10.8 (5-14)	N/A

^a^T2DM: type 2 diabetes.

^b^HbA_1c_: glycated hemoglobin.

^c^N/A: not applicable.

^d^The Problem Areas In Diabetes 5 (PAID5) is a measure of disease-specific self-efficacy that emphasizes well-being. A total score of ≥8 indicates possible diabetes-related emotional distress and warrants further clinical assessment.

Perhaps most notably, individuals in this group demonstrated improvements despite poor disease management at baseline that was significantly worse than group 1 (average HbA_1c_ of 10.0%, refer to Table 2 for complete outcome data):

On the odd occasion that my numbers get high, to have the ‘let’s retest in 3 hours’ message [pop up], you know that’s been a help as well because it helps me in managing my diabetes.

SITE C03

That was the key benefit for me. So when I started my A_1c_ was 11.1 and when I got it done, when I saw the nurse, 3 weeks ago, it was 8…A dramatic reduction, and I’m still trying to get it down, but it’s a pretty dramatic reduction for me…I don’t think I’ve had an A_1c_ of 8 for a number of years.

SITE C02

Similar to the first group, timely feedback and the ability to identify factors that trigger blood glucose spikes increased participants’ overall confidence to self-manage their T2DM. Increased confidence also served as a mechanism to resolve unwelcome emotions such as anger and frustration that participants had experienced when struggling to master self-management over the course of their condition:

Yeah. No I um, I do really like it, it’s kind of kept me going in making me feel a little bit stronger in myself and that with it...Yeah, more confident that I can do it.

SITE B02

### Group 3: Failure to Activate or Meet Needs—Low Engagers and Dropouts

The final group of C-M-O configurations concerns a group of individuals who had little to no self-efficacy before the intervention; identified a range of psychosocial issues that featured more prominently in their narrative than T2DM; reported a range of competing priorities, including work, family, and school; were uncertain of the benefits of using mobile technology to support self-management; and had a wide range of disease duration (from newly diagnosed to long-standing):

It’s just a matter of just double checking...And um, if it would connect to the foods I put in to what insulin readings I put in, that would be good, cuz right now it just seems kind of useless. Right now, it’s just a matter of double putting in my glucose readings.

SITE C05

I go back and click on that date and enter all my sugars and meds and what not [all at once]. It’s a lot easier than doing it daily—doing it daily it just eats up so much of my time. I only get a half hour lunch break at work usually…I don’t want to spend my time fussing with it.

SITE B05

Individuals in this group described their experience with managing T2DM as a struggle and viewed the diagnosis as a burden. A range of external barriers were cited that interfered with the ability to self-manage, including a sense of deprivation, unhelpful encounters with health care providers, and a hectic schedule. Participants also described a lack of recognition around proper strategies for self-management and reluctance to engage in basic self-management behaviors (eg, insulin adherence and testing blood sugar levels):

I find it hard every time to take my insulin...it’s a real chore...Yeah, and trying to find space in my stomach that doesn’t hurt...I just feeling like giving up sometimes and not taking it...The times I haven’t taken it, it uh, then I get mad at myself which doesn’t help the situation.

SITE C05

It’s not really fitting very well because I’m going to be honest I don’t really even test my sugars as much as I should. Because sometimes I will miss the time taking my insulin...I haven’t tested my sugar in a while.

SITE A04

Multiple competing priorities were highlighted, including work and caring for children. Narratives revealed a lack of responsibility for individual actions and a host of cyclical negative emotions, including anxiety, anger, and frustration. Individuals in this group did not identify members of a support system, either from their personal network or their health care team:

I don’t even know why the clinic was there, it was like, this is a complete waste of my time. You know, I already knew what she told me, like there was no help, you know there was no information offered, I left there empty handed.

SITE A02

Well the phone was giving me problems at first. So the first thing in my head was ‘uh-oh am I going to have a problem with the phone.’ And that’s when I requested if I could get another phone, but then she said we’ll try it again. She went out the room, came back in, I think the first interaction was kinda of–it sucked.

SITE C01

Participants highlighted several features of the mobile app that may have represented mechanisms of action but were not activated for this group. The project coordinators at 2 of the sites (who were responsible for introducing the intervention to participants at sites A and C) were perceived to be minimally engaged with participants. This may have represented a missed opportunity for this group of individuals in the absence of strong support networks:

I may have gotten an email but there was definitely no phone contact or anything. And I think the only contact that they reached out to me for was making sure I was going to get my [blood glucose test].

SITE A02

Overall, individuals in this group failed to integrate the mobile app into routine daily activities, and generally perceived data entry as a burden. Unlike participants in other groups, these individuals viewed the intervention as a duplication of current logbook methods (eg, handwritten) and did not perceive the technological advancement as a relative advantage:

Nah. If I forget about it, it sits in my bag, like my pill bag and you go to turn it on and it’s dead. Then you gotta plug it in, and then cause you unplug it and put it back in the bag and you forget about it again, right? It’s not in my pocket.

SITE A03

Failure to activate potential mechanisms of action is particularly relevant, as some participant narratives reveal preliminary signs of intervention potential and positive influence. Nonetheless, the mobile app failed to activate mechanisms of actions for these individuals, and their outcomes remained unchanged (refer to Table 2).

And you know [the mobile application] kinda does make you think what you should do differently, and obviously then it’s just self-management after that.

SITE A02

I go back and click on that date and enter all my sugars and meds and what not. It’s a lot easier than doing it daily—doing it daily it just eats up so much of my time. Like I only get a half hour lunch break at work usually. I don’t want to spend my time fussing with it.

SITE B05

Within the first few weeks of use, 3 individuals dropped out of the study and returned their mobile device. Participant narratives revealed that T2DM self-management was competing with prominent psychosocial issues for attention, and was therefore not a high priority. Strong negative emotions were central to each individual’s experience, and included feeling overwhelmed with the idea of change, wanting to give up, and struggling to cope. These experiences were compounded by a range of competing priorities, including family, work, and school. As a result, individuals were not motivated to better manage their T2DM:

It’s overwhelming…I’m not really depressed but I get glum […] Like I get to the point where—the hell with it—and I’ll open a can of coke because it’s more like a pissed off that I’m going through this, and maybe if I intake enough of bad stuff, I have the seizure or go into the coma or something—not that I’m suicidal or anything…

SITE B03

I feel deprived of certain things that I want to eat and I know I can’t eat it. Uh, it affects me, my mood, some days I’m happy some days I’m sad, um, it I guess that’s what triggers my depression in some ways [...] Just sometimes I get frustrated and sometimes feel like giving up.

SITE C01

These 3 participants reported limited to no use of the mobile app before dropout, precluding the ability for the intervention to influence change:

Even if I did [everything I’m supposed to], starting to use [the app] on a regular basis is gonna be hard too. Because it’s not that I’m unwilling which is partly true, I am unwilling—I shouldn’t have to do this.

SITE B03

## Discussion

### Principal Findings

The results of this study identify variations in patient characteristics that influenced the adoption and outcome of a mobile-based self-management app to improve self-management in T2DM. To our knowledge, this is the first realist evaluation to systematically link a cluster of patient-level determinants to clinical outcomes with a specified mechanism of action. The results suggest that an individual’s self-efficacy, competing priorities, evidence of previous behavior change, and beliefs about Web-based solutions interact to determine the impact on engagement and clinical outcomes. Furthermore, the balance of these characteristics may be useful for identifying individuals who may need more intensive support, informing the allocation of health care resources.

Our findings align with previous qualitative literature identifying increased awareness as a mechanism underlying successful engagement with a Web-based solution for T2DM [43]. Participants whose HbA_1c_ improved >1% reported that the intervention improved their self-efficacy to manage their diabetes, whereas those who failed to achieve these gains reported competing demands that limited engagement with intervention [43]. Self-efficacy is influenced through previous experiences of success, social persuasion and encouragement, social models of success and failure observed from individuals perceived to be similar, and stress and tension [44]. Many participants in this study described the feedback messaging as motivational and encouraging, whereas others reported frustration when glucose readings fell outside the target range and messaging failed to provide encouragement. Feedback messages were triggered in response to available blood glucose data and were not triangulated with other inputs (or lack thereof), which may present an opportunity to further tailor messaging to encourage improvements among poor performers.

Targeting outcome expectations can be easily integrated into a Web-based solution and present one strategy to regulate patient motivation when previous experiences have been unsuccessful [44]. The app included passive access to a resource library that includes a rotating assortment of videos; however, actively directing users to this content may be required for those who require additional support. The current version also included 3 levels of tailored messaging (from a beginner level to more sophisticated content); however, all 3 levels addressed the full range of self-management issues. Interventions designed to promote incremental knowledge gain and experiential and vicarious learning are better positioned to impact individual ability to self-manage [45], suggesting that a graduated approach introducing a few concepts at a time may be more beneficial when implementing complex interventions targeting behavior change. Many individuals with poorly controlled diabetes are not sufficiently confident or motivated to initiate and maintain self-management changes [46], emphasizing the need for mobile self-management apps to explicitly target readiness to change and emphasize increasing self-efficacy to optimize the potential for impact.

A systematic review of mobile apps for diabetes management found 5 of the 6 studies reported positive feedback on usability and feasibility, whereas only 3 reported statistically significant reductions in clinical outcomes such as HbA_1c_ and blood pressure [47]. These heterogeneous findings demonstrate that positive patient feedback does not always accompany clinical improvements, highlighting that a range of factors interact to contribute to a successful impact on outcomes. This underscores the need to identify patient and intervention characteristics that are likely to facilitate both outcomes. Whether and how a Web-based solution enables or limits the possibility for relationships with professionals, the degree of fit with participants’ everyday life and capacity, and pattern visualization of symptoms and feedback are key mechanisms to support successful implementation [48]. Our results also suggest that the impact of Web-based solutions would be enhanced if they were equipped with the ability to adapt to individual users based on the triangulation of available data and proactively identify individuals who require additional support to avoid disengagement.

Among many individuals who failed to achieve engagement and a reduction in HbA_1c_ (group 3), the results provide 2 key insights. First, triangulation with quantitative outcomes (ie, the PAID5) reveals average borderline emotional distress among low engagers and dropouts that warrants further clinical examination. Among low engagers, emotional distress increased slightly from baseline (average PAID5 score of 10.0) to 3 months (average score of 10.8), suggesting it was not effectively addressed as part of the participants’ ongoing care. Diabetes-related emotional distress is significantly related to HbA_1c_ levels [49], underscoring the importance of effectively targeting distress to achieve improvements in glycemic control. This could be achieved through more targeted clinical care or by exploring opportunities to address emotional distress as part of a comprehensive Web-based solution. Second, the results reveal an opportunity to modify the Web-based solution or its implementation to address currently unmet needs. Low perceived value and a lack of patient-provider interactions are barriers to engaging in Web-based solutions for T2DM [50,51]. Health literacy is also likely to affect self-management behaviors [52] and is lower among disengaged patients, indicating the need to address underlying cognitive and social skills that determine an individual’s motivation and ability to understand and use information to inform healthy behaviors. Similar to our study findings, Lie et al [51] identified that prioritizing other activities and frustrations with the technology led to a loss of motivation among dropouts of a Web-based solution for T2DM. Individuals with T2DM exhibit a variety of dominant coping mechanisms; however, those exhibiting problem-focused and avoidance-focused mechanisms are significantly less likely to be adherent to self-care activities [53]. Web-based solutions can leverage existing measures to evaluate coping strategies to identify individuals who are at risk of suboptimal adherence to inform the provision of additional support(s) as needed.

Individuals manage chronic conditions within different (but not exclusive) nonprofessional contexts where relationships are primarily patterned and unreflexive [54]. Reeves et al [55] demonstrated that health service costs were significantly reduced for individuals who experience greater levels of illness work (eg, crisis prevention and management, symptom management, and disease-specific activities) through their social networks. Illness work was associated with increased self-management, healthy behaviors, and emotional well-being [55], underscoring the value of both harnessing and sustaining the potential of social networks to support the success of self-management interventions. The Web-based solution did not incorporate a social function, nor did the implementation include a mechanism to integrate the solution into the existing social networks. Improvements in clinical outcomes in this study may have been mediated by strong social networks, as these individuals were able to successfully incorporate the solution into the existing routines and negotiate competing priorities. In contrast, individuals who were unsuccessful or disengaged did not identify a pre-existing source of social support. The influence of social mechanisms on individual success should be considered in the design and implementation of Web-based self-management solutions, aligning with the growing recognition that self-management must move beyond an individual-centered view to consider the broader social context [54,56].

Finally, health care provider feedback enhances the impact of mobile self-management apps on HbA_1c_ reduction [22], underscoring the importance of active provider engagement as a support strategy in the early stages of implementation, tailoring support provision throughout the process. In this study, failure to actively engage health care providers during implementation may have contributed to a lack of sustained engagement among those participants for whom the Web-based solution failed to activate change. Further work is needed to understand whether adaptations to the Web-based solution or its implementation would have resulted in different outcomes for these individuals in their contexts. Our findings suggest that targeting outcome expectations, addressing diabetes-related emotional distress, including content to address health literacy, tailoring messaging according to individual coping strategies, and leveraging social networks are worthwhile components to consider as part of a Web-based solution.

### Limitations

Participation in the qualitative interviews was voluntary, which introduces the potential of selection bias. To mitigate this, purposive sampling was used to capture the perspectives of participants who had minimal engagement with the Web-based solution. The transferability of the results is limited by the inclusion of a small number of participants across 3 recruitment sites in a confined geographical area. The inclusion of a small number of participants and sites was necessary to achieve a depth of understanding with respect to contextual factors, the features of the Web-based care solution, and how these relate to outcomes. The findings of this study serve as a foundation for future research aimed at achieving a broader understanding of how Web-based solutions work for different patients in a variety of health care contexts. Given the lack of health care provider interaction, it would be beneficial to supplement patient perspectives with those of their health care providers. Finally, a nuanced exploration of the impact of social networks was beyond the scope of this study. Given the strong influence of competing priorities as a contextual factor and the pattern of social support across groups, further work is needed to understand the extent to which both formal and informal social networks play a role in mediating the adoption of self-management behaviors and engagement with the intervention, which may in turn influence clinical outcomes.

### Conclusions

An individual’s self-efficacy, competing priorities, prior success with behavior change, and beliefs about their health interact to determine engagement with a mobile app to self-manage T2DM and its impact on clinical outcomes. Careful consideration of the balance of these characteristics is likely to help health care providers identify individuals who are more likely to benefit from a Web-based solution and identify those requiring more intensive support and clinical resources. Web-based solutions could also be optimized to support tailored care, including the incorporation of the existing readiness- and risk-assessment measures, to assist in identifying individuals who are at risk of suboptimal adherence to inform the provision of additional support(s) as needed.

## Appendix

Multimedia Appendix 1 Interview guide.

## Abbreviations

C-M-O: context-mechanism-outcome
ITG: immediate treatment group
OTN: Ontario Telemedicine Network
PAID5: Problem Areas In Diabetes 5
T2DM: type 2 diabetes
WLC: wait-list control group
