# Supplementary material for: A Mobile App to Improve Self-Management of Individuals With Type 2 Diabetes: Qualitative Realist Evaluation
Source: J Med Internet Res. 2018 Mar 16;20(3):e81. doi: 10.2196/jmir.8712 (PMC5878364; doi:10.2196/jmir.8712)
Supplement: Multimedia Appendix 1 [file jmir_v20i3e81_app1.pdf]

Appendix: Patient/Caregiver Interview Guides

Perceived Needs and Concerns

1. In your current situation, can you identify what your health care needs are? (i.e. support services, education, equipment)

    At Follow-Up: How have your needs changed over the past 6 months? (i.e. when you started to use the BluStar application)

2. Can you describe any concerns you have about your health and general well-being?

Operational Characteristics: How do participants interact with and experience the BluStar application?

1. Can you describe how you have used the BluStar application (e.g. where you were when you logged on, who you were with, how often)?

    a. What features were you able to use through the application (specifically probe use of carb counter, activity tracking, restaurant feature, etc.)?
    b. How did you learn how to use these features?
    c. What did you think of the design and layout?

2. How well were you able to use the application as part of your daily life? Can you describe how this became part of your routine (or why it did not)?

3. How was the Smart Visit report incorporated into your treatment plan?
    a. Who received it, used it and how was it used?

4. How did the BluStar application help support you in following the recommendations of your healthcare team and managing your diabetes?

    Prompts:
    a. What aspect(s) of the application do you think worked well/did you like to help you better manage diabetes?
    b. What aspect(s) of the application did not work well/did you not like?
    c. What aspect(s) of the application could we change to make it more effective?
    d. How did the using the application make you feel?

5. Do you have access to other ways (apps, devices, paper charting) to help you manage your diabetes?

    At Follow-Up: What is the value of this application compared to other options available to you?

6. Is there anything else we could have done to better support you in using BluStar Application?

Mechanism/Outcome: Explore how the BluStar application impacted diabetes self-management

1. Have you noticed any changes in yourself since using the application?
   Prompts:
   - Changes to your health, mood or confidence, improved memory, relationships with healthcare providers, improved care experience

2. What do you think happened that led to those changes?
   Prompts:
   - Did it improve what you knew about treatment options/your condition?
   - Did it improve your ability to self-monitor?
   - Did it change your outcome expectations, your priorities, or your intentions?

3. What motivates you to use the app?

4. What keeps you from using it/using it more?

5. What do you need to better manage diabetes?

Questions for Follow-Up

Contextual Characteristics: How do individual attitudes and experiences influence perceptions of the BluStar application? (review previous transcribe for contextual information related to the participant)

1. Has your comfort level with using the BluStar application (or other technology) for managing your diabetes changed?

2. You mentioned **[xxxx]** enablers associated with using the application in your first interview. Have these changed? Please describe.

3. You mentioned **[xxxx]** challenges associated with using the application in your first interview. Have these changed? Please describe.

4. Would you recommend the BluStar application to a patient with similar needs as you? Why?

5. What suggestions would you have for a mobile application if it was going to be offered across Ontario? What would you do differently?

6. Is there anything else that you would like to mention we haven’t discussed today?
